# Chromatin dynamics associated with seed desiccation tolerance/sensitivity at early germination in *Medicago truncatula*

**DOI:** 10.3389/fpls.2022.1059493

**Published:** 2022-11-24

**Authors:** Naoto Sano, Jaiana Malabarba, Zhijuan Chen, Sylvain Gaillard, David Windels, Jerome Verdier

**Affiliations:** Univ Angers, Institut Agro, INRAE, IRHS, SFR QUASAV, Angers, France

**Keywords:** seed germination, desiccation tolerance, chromatin, ATAC-Seq, ChIP-Seq, H3K27me3, *Medicago truncatula*, H2AK119ub

## Abstract

Desiccation tolerance (DT) has contributed greatly to the adaptation of land plants to severe water-deficient conditions. DT is mostly observed in reproductive parts in flowering plants such as seeds. The seed DT is lost at early post germination stage but is temporally re-inducible in 1 mm radicles during the so-called DT window following a PEG treatment before being permanently silenced in 5 mm radicles of germinating seeds. The molecular mechanisms that activate/reactivate/silence DT in developing and germinating seeds have not yet been elucidated. Here, we analyzed chromatin dynamics related to re-inducibility of DT before and after the DT window at early germination in *Medicago truncatula* radicles to determine if DT-associated genes were transcriptionally regulated at the chromatin levels. Comparative transcriptome analysis of these radicles identified 948 genes as DT re-induction-related genes, positively correlated with DT re-induction. ATAC-Seq analyses revealed that the chromatin state of genomic regions containing these genes was clearly modulated by PEG treatment and affected by growth stages with opened chromatin in 1 mm radicles with PEG (R1P); intermediate openness in 1 mm radicles without PEG (R1); and condensed chromatin in 5 mm radicles without PEG (R5). In contrast, we also showed that the 103 genes negatively correlated with the re-induction of DT did not show any transcriptional regulation at the chromatin level. Additionally, ChIP-Seq analyses for repressive marks H2AK119ub and H3K27me3 detected a prominent signal of H3K27me3 on the DT re-induction-related gene sequences at R5 but not in R1 and R1P. Moreover, no clear H2AK119ub marks was observed on the DT re-induction-related gene sequences at both developmental radicle stages, suggesting that silencing of DT process after germination will be mainly due to H3K27me3 marks by the action of the PRC2 complex, without involvement of PRC1 complex. The dynamic of chromatin changes associated with H3K27me3 were also confirmed on seed-specific genes encoding potential DT-related proteins such as LEAs, oleosins and transcriptional factors. However, several transcriptional factors did not show a clear link between their decrease of chromatin openness and H3K27me3 levels, suggesting that their accessibility may also be regulated by additional factors, such as other histone modifications. Finally, in order to make these comprehensive genome-wide analyses of transcript and chromatin dynamics useful to the scientific community working on early germination and DT, we generated a dedicated genome browser containing all these data and publicly available at https://iris.angers.inrae.fr/mtseedepiatlas/jbrowse/?data=Mtruncatula.

## Introduction

Desiccation tolerance (DT) can be defined as the ability of an organism to survive drying to about 10% remaining water content, which is roughly equivalent to 50% relative air humidity at 20°C and to dropping to a water potential of –100·MPa (Alpert, 2006). This threshold clearly separates desiccation sensitive (DS)- from DT-species (Alpert, 2005). DT is an ancient trait in plants and is almost universal in land plants, but it is mostly confined to reproductive structures such as spores, pollen or seeds, and only a few species having DT in vegetative tissues such as resurrection plants (Oliver et al., 2020). Studies on resurrection plants showed that they possess the ability to activate DT mechanisms similar to seeds in their vegetative tissues in response to water losses (Costa et al., 2017; VanBuren et al., 2017). In angiosperms, most species have desiccation tolerant seeds and/or pollen, therefore possess the genetic information for DT (Blomstedt et al., 2018). However, our understanding of molecular mechanisms how the DT is activated in the reproductive structures and repressed in vegetative tissues still remains unclear.

The majority of angiosperm species produce seeds termed “orthodox seeds” that are DT and long-term dry storable (Roberts, 1973). The orthodox seeds acquire DT during seed development at early maturation phase (Ooms et al., 1993; Ellis and Hong, 1994) and the timing of DT acquisition is a highly stable trait, that is not affected by abiotic stresses, such as heat and osmotic stresses (Righetti et al., 2015). After seed germination, seeds lose the DT and subsequently established desiccation sensitive seedlings. Nevertheless, at early germination during the loss of DT, there is a developmental window (DT window) during which DT can be re-induced. In *Medicago truncatula*, a model Fabaceae, the re-induction of DT is observed following a mild osmotic stress treatment using polyethylene glycol (PEG) on germinated seeds (*i.e.*, 1 to 3 mm of protruded radicle length) but the DT is no longer re-inducible at later seed germinating stage (*i.e.*, 5 mm of radicle length) (Buitink et al., 2003; Faria et al., 2005; Buitink et al., 2006), indicating the timing of this DT window. This DT window was also observed in different species including *Cucumis sativus, Impatiens walleriana, Tabebuia impetiginosa* and *Arabidopsis thaliana* (Bruggink and van der Toorn, 1995; Vieira et al., 2010; Maia et al., 2011). These results suggest that although DT is programmed to be suppressed at early post-germination phase, there is a universal mechanism that allows its re-induction following cues from the external environment (*e.g.*, mild osmotic stress) within the DT window. The differences in DT inducibility within and without the DT window can be used as a model biosystem for understanding molecular mechanisms that activate/repress DT in specific plant tissues.

The molecular processes involved in DT acquisition and re-induction have been well studied at the transcriptomic level, which resulted in identification of DT-associated gene networks. These DT-associated genes were mainly associated with cell protection functions (*e.g.*, antioxidants and protective compounds), promoting cell survival upon severe dehydration (Delahaie et al., 2013; Terrasson et al., 2013; Verdier et al., 2013; Costa et al., 2015; Righetti et al., 2015; González-Morales et al., 2016; Costa et al., 2017; Bizouerne et al., 2021). These DT-associated gene networks are transcriptionally regulated at potentially two levels: a more explored direct transcriptional regulation *via* the action of transcriptional activators/repressors such as transcription factors and/or at the epigenetic level *via* the regulation of the chromatin accessibility rendering the DT-associated genes in a repressed or activable states. However, the nature of the precise regulatory mechanisms of how these DT-related networks are specifically activated or repressed in plant DT/DS tissues are still largely unknown and the chromatin accessibility of DT-associated genes has never been checked to our knowledge.

Indeed, chromatin accessibility was directly associated with gene expression levels and specific developmental functions or reprogramming (Jung et al., 2017). Moreover, it has been shown that Polycomb group complexes were involved in gene silencing by switching from genomic regions from an opened to condensed chromatin state (King et al., 2018; Yin et al., 2021). Both Polycomb Repressive Complex1 (PRC1) and Polycomb Repressive Complex2 (PRC2), originally identified in Drosophila, are essential in initiating and/or maintaining genes in repressive status by epigenetically modifying chromatin dynamic in plants, but act differently (Hinsch et al., 2021). PRC2 silences gene expression by trimethylating Lys27 of histone H3 (generating H3K27me3). In response to H3K27me3, PRC1 mono-ubiquitylates Lys119 of histone H2A (generating H2AK119ub), further silencing the genes already repressed by PRC2 (Schwartz and Pirrotta, 2013). Although, the timing of this silencing process is still discussed as it has been shown that PRC1 may act earlier than PRC2 (Yang et al., 2013). Several models have proposed that the PRCs and/or their associated histone modifications H3K27me3 and H2AK119Ub establish chromatin compaction, which directly or indirectly inhibits RNA polymerase II, resulting in gene transcriptional repression (Aranda et al., 2015). The mechanism of transcriptional repression and three-dimensional structure of chromatin regulated by PRCs is still enigmatic and has not been fully elucidated (van Mierlo et al., 2019). Recently, chromatin accessibility profiling in plants using Assay for Transposase-Accessible Chromatin using sequencing (ATAC-Seq) has been employed in many species and has revealed a wealth of new information regarding the regulatory structure and dynamics of plant genomes (Bubb and Deal, 2020). In the context of epigenetic regulation of seed maturation, dormancy, and germination, different types of histone post-translational modifications have been described to date (Lepiniec et al., 2018; Smolikova et al., 2021; Ding et al., 2022). It is thus possible that DT window during seed germination is also regulated at the level of histone modifications and chromatin remodeling.

Here, we performed ATAC-Seq and ChIP-Seq of specific histone marks associated with PRC1 (H2AK119ub) and PRC2 (H3K27me3) on post-germinated *Medicago truncatula* radicles showing different DT re-inducibilities, aiming to know the epigenetic regulatory mechanisms of DT re-induction of germinating seeds at chromatin levels.

## Results

### Re-induction of DT to Medicago seeds at early post germination

**Figure 1:**
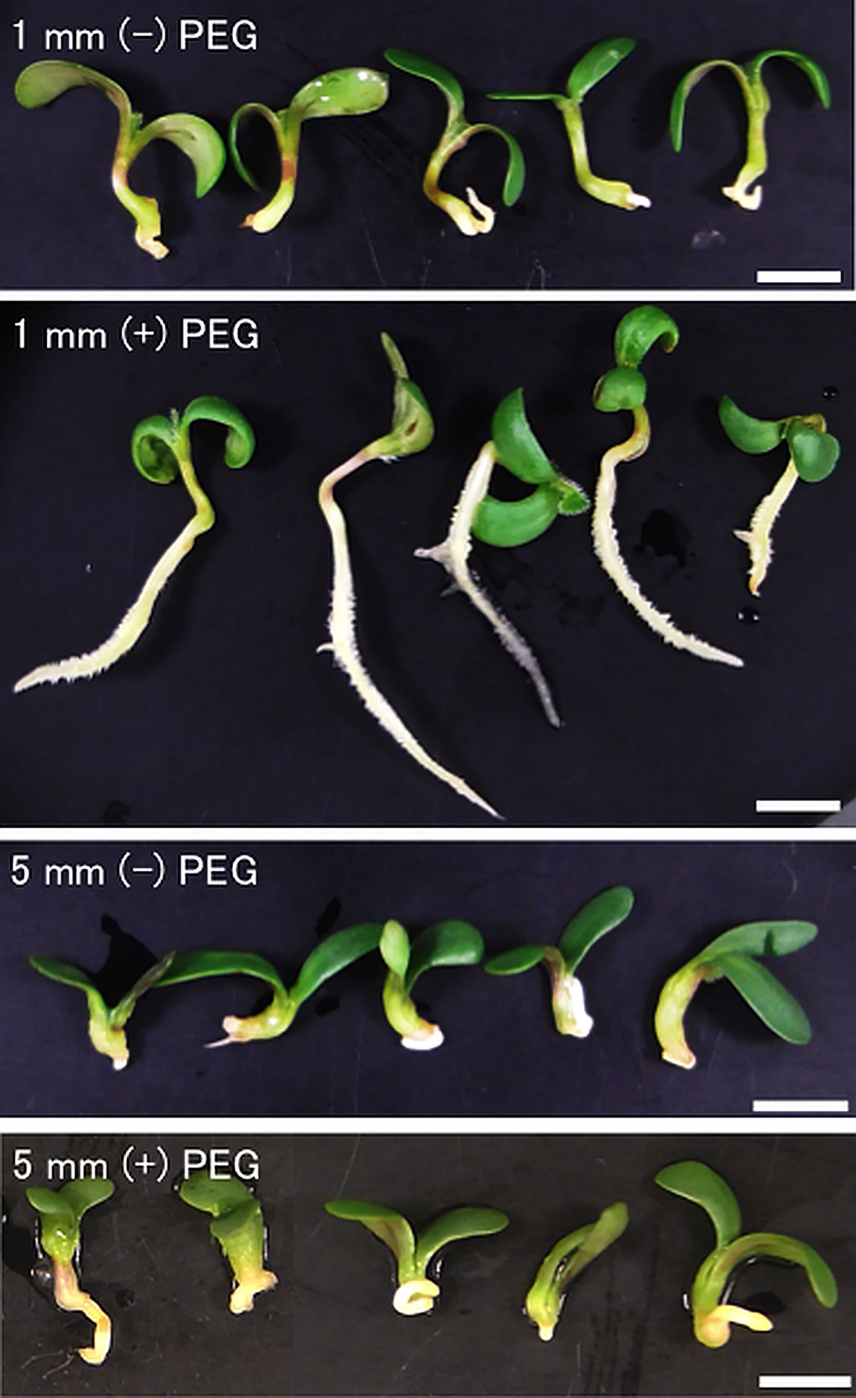
Inducibility of radicle desiccation tolerance by PEG treatment in germinating seeds of *Medicago truncatula.* Seeds upon germination were selected using radicle length. Germinating seeds displaying 1 mm or 5 mm radicles were either directly desiccated (R1, R5) or treated with a mild osmotic stress by PEG8000 (R1P, R5P), followed by desiccation treatment. After desiccation, differences in radicle growth were observed at 7 days after rehydration. White bar scales represent 5 mm.

To confirm the re-inducibility of DT in germinating seeds by PEG treatment, post-germinated seeds displaying 1 mm and 5 mm radicles were both treated by PEG or untreated, then dried and re-hydrated. After 7 days, we observed that 1 mm radicle seeds treated by PEG showed healthy seedlings but instead 1 mm radicle seeds untreated with PEG did not resume root growth after the desiccation process (**Figure 1**). This result indicated that PEG treatment corresponding to a mild osmotic stress on seeds with 1 mm radicle seeds was able to re-induce the DT program, whereas non-treated 1 mm radicle seeds failed to re-induce it (**Figure 1**). Regarding the 5 mm radicle seeds, we observed that with or without PEG treatment, seeds did not re-induce root growth and therefore confirming that DT is not re-inducible in early post germinating seeds after a specific stage marking the end of the DT window as reported previously (Faria et al., 2005). With regard to cotyledons, a relatively high proportion of healthy and green cotyledons were observed in 1 mm and 5 mm radicle seeds subjected or not to PEG treatment, which is consistent with previous findings that cotyledon tissues are more tolerant to desiccation stress than other germinating tissues (Faria et al., 2005; Maia et al., 2011). Based on this assay, we collected 1- and 5-mm radicle tissues subjected or not to PEG treatment. In the following part of this study, we will focus on the radicle tissues with 1 mm radicles PEG-treated (R1P), which were DT tissues and compared it to untreated 1 mm and 5 mm radicles (R1 and R5), which were DS tissues (**Table 1**).

**Table 1:** Abbreviations of samples used in this study.

Sample	Abbreviation	Phenotype
Radicle 1 mm without PEG	R1	DS
Radicle 1 mm with PEG	R1P	DT
Radicle 5 mm without PEG	R5	DS
Radicle 5 mm with PEG	R5P	DS

### Expression pattern of DT-inducible genes

**Figure 2:**
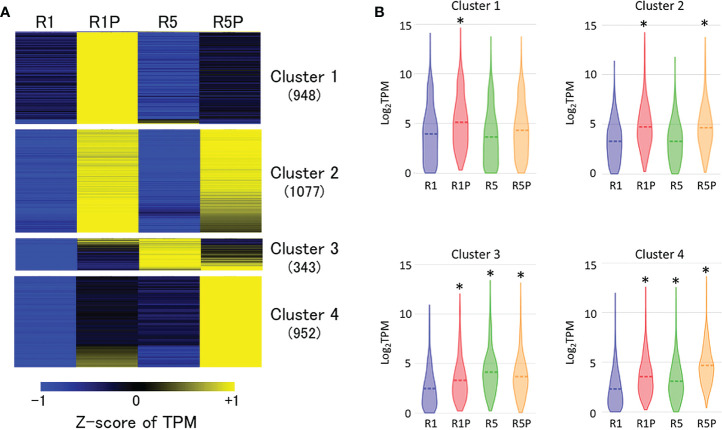
Expression patterns of genes induced by PEG treatment. **(A)** K-means cluster analysis of 3,337 genes whose expression is significantly up-regulated at R1P compared to R1 (adjusted *P* < 0.05; Benjamini–Hochberg method, log_2_ fold change > 1). The number in the parentheses indicates the number of genes in each cluster. Z-score of TPM values was used to normalize each gene expression level. **(B)** Violin plots showing log_2_TPM values of genes in each cluster at R1, R1P, R5 and R5P (*P < 0.01, Steel–Dwass test, compared to R1). Dashed lines represent the mean value for each sample.

Aiming to identify a gene cluster involved in PEG-induced DT, RNA-Seq of R1, R1P, R5 and R5P samples was performed. Comparative transcriptome analysis revealed 7,065 differentially expressed genes (DEGs) between R1 vs R1P (adjusted *P* < 0.05; Benjamini–Hochberg method, |log2 fold change| > 1) in which 3,337 genes were up-regulated at R1P. The up-regulated genes were then classified into four clusters based on expression values (Z-score of TPM) at R1, R1P, R5 and R5P samples using K-means cluster analysis (**Figure 2A**; **Supplementary Table S1**). Genes in cluster 1 were significantly up-regulated only in R1P (*P* < 0.01, Steel–Dwass test) (**Figure 2B**), which corresponded to the only DT tissue (**Figure 1**; **Table 1**). Therefore, genes from cluster 1 were potentially involved in the re-induction of DT by PEG treatment. Cluster 2 genes were significantly increased in both R1P and R5P but not R5 indicating that these genes were potentially PEG-responsive genes. We considered cluster 2 genes not conclusively associated with the re-induction of DT, although some may contribute to the basal levels of DT. Both cluster 3 and 4 genes showed increases at R1P, R5 and R5P, with cluster 3 and 4 genes being most prominently induced at R5 and R5P respectively, and therefore not associated with DT re-induction.

### ATAC-Seq profiles of DT-inducible genes

**Figure 3:**
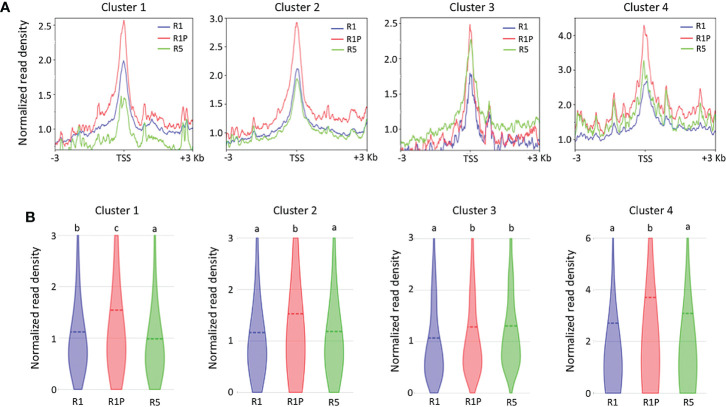
Differences in chromatin accessibility of genes belonging to each cluster among R1, R1P and R5. **(A)** TSS enrichment scores from ATAC-seq were plotted at ±3 Kb of the transcription start site (TSS) of genes in each cluster. **(B)** Violin plots showing normalized read density of genes (1 Kb promoter + mRNA) in each cluster at R1, R1P and R5. Dashed lines represent the mean value for each sample. Different letters indicate significant differences (*P < 0.01, Steel–Dwass test).

To clarify if the DT re-induction in germinating seeds is regulated at the level of chromatin accessibility or/and *via* more direct transcriptional regulation (*e.g. via* regulation through transcription factors), we performed ATAC-Seq on R1 and R5 as DS samples and on R1P as a DT sample. Then, we compared the chromatin accessibility by analyzing the transcription start site (TSS) enrichment score of all genes contained into the four clusters (**Figure 3A**). At R1P, chromatin accessibility was significantly increased with respect to R1 across all four clusters (*P* < 0.01, Steel–Dwass test) (**Figure 3B**), consistent with the fact that transcript levels of these genes were up-regulated at R1P by PEG treatment (**Figure 2B**). This result also suggested that many gene expressions induced by PEG treatment on the early phase of post-germinated seeds were regulated by the level of chromatin accessibility. Regarding R5 samples, many levels of chromatin accessibility were detected with respect to different clusters. Interestingly, chromatin of genes belonging to cluster 1, which is associated with DT re-induction, was more accessible in R1 than in R5 (**Figure 3B**), although there was no significant difference at their transcript levels (**Figure 2B**). For cluster 4 genes, the transcript level was significantly up-regulated at R5 than R1 whereas the accessibility of chromatin was comparable. Other clusters 2 and 3 showed consistent patterns between expression and chromatin accessibility levels. These results clearly indicated that R1 represents an intermediate state regarding chromatin openness level of cluster 1 genes, potentially involved in DT re-induction. Whereas, in R1P, chromatin regions of cluster 1 genes were more accessible, suggesting a potential transcriptional expression, whereas in R5 chromatin regions of cluster 1 genes were tightly condensed and non-accessible. The chromatin accessibility levels within the entire genome were performed in all three stages (*i.e.*, R1, R1P and R5) in this study and these data are publicly available on a dedicated genome browser (https://iris.angers.inrae.fr/mtseedepiatlas/jbrowse/?data=Mtruncatula).

### ChIP-Seq profiles of genes related to DT re-induction

**Figure 4:**
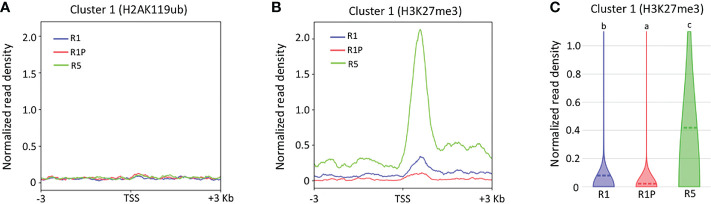
Differences in histone marks of genes belonging to clusters 1 among R1, R1P and R5. TSS enrichment scores from ChIP-Seq of H2AK119ub **(A)** and H3K27me3 **(B)** were plotted at ±3 Kb of the transcription start site (TSS) of genes belonging to cluster 1. **(C)** Violin plots showing normalized read density of ChIP-Seq (H3K27me3) for genes (1 Kb promoter + mRNA) of cluster 1 at R1, R1P and R5. Dashed lines represent the mean value for each sample. Different letters indicate significant differences (*P* < 0.01, Steel–Dwass test).

To better understand the molecular mechanisms by which chromatin accessibility is regulated during DT re-induction (cluster 1), ChIP-Seq of specific histone marks H2AK119ub and H3K27me3 were performed on the same samples as ATAC-Seq and TSS enrichment score was calculated for genes from the cluster 1. No clear signal was observed for H2AK119ub marks (**Figure 4A**), which suggested that cluster 1 genes were not bound to the H2AK119Ub histone marks in any of the three stages (*i.e.*, R1, R1P and R5). Similarly, no clear signal was observed for H3K27me3 histone marks within the genomic regions of cluster 1 genes in R1 and R1P, whereas a very strong signal within the same regions was observed in R5 (**Figure 4B**). Statistical analysis confirmed that the H3K27me3 marks were significantly lower in R1P and prominently higher in R5 compared to R1 (*P* < 0.01, Steel–Dwass test) (**Figure 4C**), being consistent with the pattern of chromatin accessibility (*i.e.*, R5 exhibited more closed chromatin, followed by P1 and finally R1P was the most accessible chromatin levels). First, these results suggested that PRC2 (H3K27me3) rather than PRC1 (H2AK119ub) was involved in the silencing of genes related to DT re-induction at the end of the DT window in R5. Moreover, it showed that PRC2 and the H3K27me3 marks were not clearly involved in repressing DT-related genes in R1 and R1P. The enrichment of genomic regions linked to H3K27me3 and H2AK119Ub histone marks were measured within the entire genome in all three stages (*i.e.*, R1, R1P and R5) and a statistical procedure to identify enriched genomic regions after immunoprecipitation with these histone marks (*i.e.*, peak calling) was performed using Macs2 software. These data are publicly available on a dedicated genome browser (https://iris.angers.inrae.fr/mtseedepiatlas/jbrowse/?data=Mtruncatula).

### Biological processes involving DT re-induction-related genes with different chromatin accessibility

**Figure 5:**
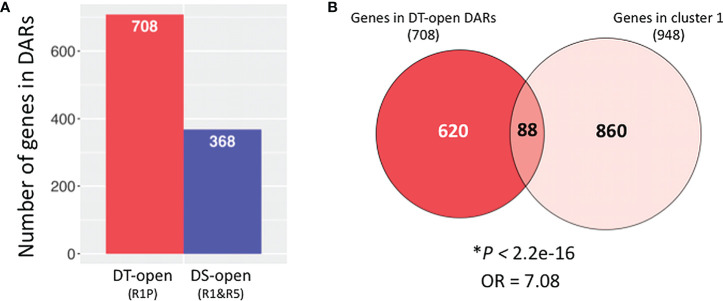
Identification of genes in differentially accessible chromatin regions (DARs) between DT and DS germinating radicles. **(A)** Number of genes (1 Kb promoter + mRNA) present in DARs between DS (R1&R5) and DT (R1P) samples (adjusted *P* < 0.05; Benjamini–Hochberg method). **(B)** Venn diagrams show overlaps between genes in DT-DARs detected by ATAC-seq and genes belonging to cluster 1 detected by RNA-seq. Asterisks indicate significant overlap (*P* values from Fisher’s exact test; OR: odds ratio which represents the strength of association, with OR>1 corresponding to a strong association).

In order to reveal what biological processes are particularly regulated at chromatin levels during the DT re-induction, we identified genes located in significantly differentially accessible chromatin regions (DARs). Comparative ATAC-Seq analysis detected 1,076 genes that were located in DARs between DS (R1&R5) vs DT (R1P) samples (adjusted *P* < 0.05; Benjamini–Hochberg method), in which 708 gene regions were more opened in DT samples (**Figure 5A**; **Supplementary Table S2**). The 708 genes in DT-DARs were then compared to the 948 genes of cluster 1 detected by RNA-Seq experiments (**Figure 5B**). As a result, we observed a significant overlap between these two datasets (*i.e.*, DEGs and DARs between DT and DS tissues) using Fisher’s exact test (*P* < 2.2E-16, OR = 7.08), indicating that cluster 1 genes are strongly associated with DT-DAR-genes. From this comparison, we highlighted 88 genes both transcriptionally activated and located in chromatin regions that are significantly more accessible in DT tissues. We herein named the 88 common genes as DT-DAR-DEGs or DT-(open)DAR-DEGs (**Supplementary Table S3**). On the other hand, we also detected 860 genes that were transcriptionally activated but not located in differential accessible regions in DT tissues, which we hereafter referred to as DT-nonDAR-DEGs (**Supplementary Table S4**). It should be noted that the DT-nonDAR-DEGs include *MtABI5*, an *Arabidopsis* bZIP transcription factor *ABI5* orthologue that is key regulator of ABA signaling, and essential for the re-establishment of DT during germination (Terrasson et al., 2013). This result suggested that *MtABI5* may be important for the DT re-induction in Medicago but its activation is regulated at the level of direct transcriptional regulation (*e.g*. by transcription factors) rather than through chromatin dynamics.

**Figure 6:**
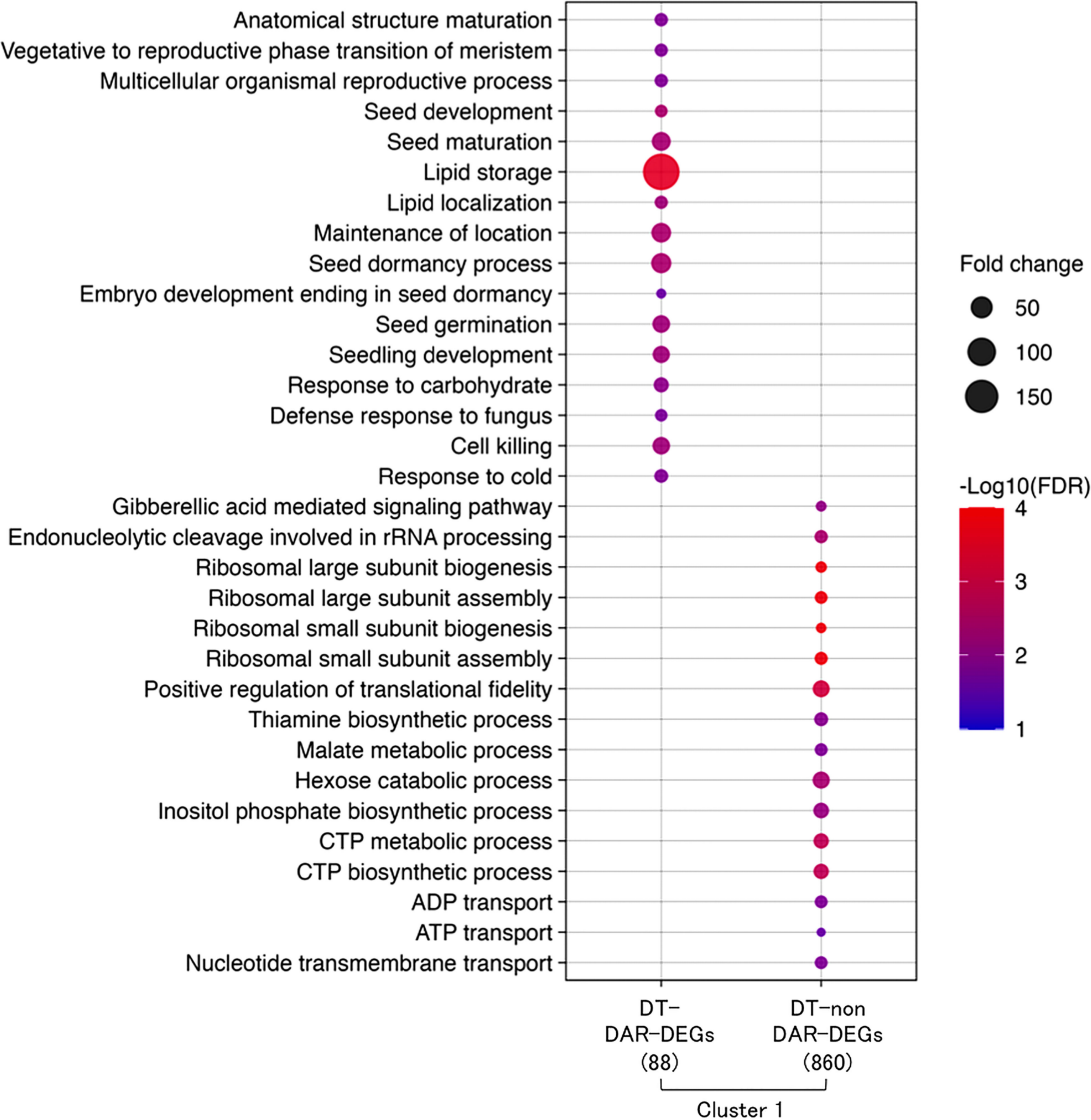
Overview of hypotheses regarding chromatin dynamics that regulates gene expressions of DT re-induction at early germination in *Medicago truncatula*. R1: germinated seeds with 1 mm radicles, R1P: germinated seeds with 1 mm radicles treated by PEG, R5: germinated seeds with 5 mm radicles, R5P: germinated seeds with 5 mm radicles treated by PEG. DS and DT indicate each phenotype of radicle (i.e., desiccation sensitive (DS) or tolerant (DT)).

A comparative gene ontology (GO) enrichment analysis was performed for the 88 DT-DAR-DEGs and the 860 DT-nonDAR-DEGs (**Figure 6**) and revealed that enriched GO terms from the two gene lists were highly contrasted, suggesting that some specific biological processes for re-induction of DT is activated first by chromatin accessibility regulation and others are turned on through more direct transcriptional regulation. The enriched GO terms of DT-DAR-DEGs were mainly related to seed development/germination, lipid metabolisms and defense responses, suggesting that gene expressions related to these biological processes were firstly regulated at chromatin levels during the DT re-induction in germinating seeds. The major GO terms of DT-nonDAR-DEGs were related to plant hormone gibberellic acid (GA) signaling, ribosome biogenesis and nucleotide metabolism/transport, indicating activation of genes for these biological pathways is more depended on transcriptional regulation rather than chromatin dynamics.

### Chromatin accessibility and histone modification profiles in representative genes of DT-DARs-DEGs

**Figure 7:**
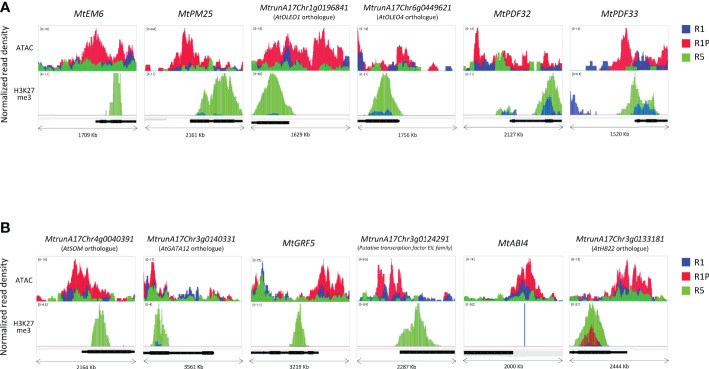
Differences in chromatin accessibility and H3K27me3 marks of DT-DAR-genes among R1, R1P and R5. Normalized read density of representative genes (1 Kb promoter + mRNA) was visualized using the Integrated Genome Viewer (IGV) for *LEA*s, *OLEO*s and *PDF*s **(A)** and transcriptional factors **(B)**. The black bar below each panel indicates the position of the respective mRNA sequence. The range in square brackets indicates the range of normalized read counts.

To understand how these DT-DAR-DEG genes were regulated at the chromatin levels, we selected some representative genes to visualize them using the Integrated Genome Viewer regarding the chromatin accessibility and H3H27me3-binding profiles (**Figure 7**). Three *late embryogenesis abundant proteins* (*LEAs*) (*MtrunA17Chr1g0205331*, *MtrunA17Chr4g0007001* and *MtrunA17Chr8g0348171*) were included in the 88 DT-DAR-DEGs (**Supplementary Table S3**), of which two *LEA*s (*MtEM6* and *MtPM25*) were plotted as examples of genes related to seed development/abiotic stress response (**Figure 7A**). Seed characteristic lipid metabolism-related genes, including four *PUTATIVE OLEOSIN*s (*MtrunA17Chr1g0196841*; Arabidopsis *AtOLEO1* orthologue, *MtrunA17Chr6g0449621*; *AtOLEO4* orthologue*, MtrunA17Chr2g0307331* and *MtrunA17Chr3g0139191*) and *PUTATIVE DIACYLGLYCEROL O-ACYLTRANSFERASE* (*MtrunA17Chr2g0299671*; *AtTAG1* orthologue), were also detected from the DT-DAR-DEGs and two of them *AtOLEO1* and *AtOLEO4 orthologues* were plotted for the profiles as representatives. Regarding defense responses, two of three *PLANT DEFENSINs* (*PDF*s) (*MtrunA17Chr2g0317741*, *MtrunA17Chr2g0317751* and *MtrunA17Chr8g0385301*) from the DT-DAR-DEGs were also plotted as example in **Figure 7A**. These plots allowed us to confirm the similar tendency at each gene level as in the chromatin/histone modification profiles of the entire gene cluster, *i.e.*, R1P showed more open chromatin than R1 and R5, and R5 displayed more prominent H3K27me3 marks. Moreover, regions of open chromatin showed a broad distribution from the promoter of the gene beyond the TSS, whereas the distribution of the H3K27me3 peaks was detected at the beginning of the gene coding sequences. Among the 88 DT-DAR-DEGs, ten genes are annotated as transcription factors (TFs) controlling expression levels of multiple target genes. We, therefore, also visualized the chromatin accessibility and H3K27me3 profiles of representative TFs (**Figure 7B**). Regarding the chromatin accessibility, all TFs showed a pattern consistent with the aforementioned *LEA*s, *OLEOSIN*s and *PDF*s, while several TFs demonstrated different patterns for H3K27me3 such as with no clear marks for *MtABI4* and higher signals in R1P than R1 for *AtHB22* orthologue. These findings suggested that the chromatin accessibility of genes related to the re-induction of DT in germinating seeds is more commonly linked to the level of H3K27me3 regarding the loss of DT in R5 tissues but the accessibility may also be regulated partially by H3K27me3 and/or by additional factors, such as other histone modifications, regarding DT re-induction between R1 and R1P.

### Genes whose expression is negatively correlated with DT re-induction

In the aforementioned series of analyses, we focused on a gene set displaying up-regulation during DT re-induction as direct markers of DT activation. However, genes repressed during the re-induction of DT may also play an important role to shut down some processes negatively correlated to DT. Therefore, we identified and analyzed the expression and chromatin dynamics of genes down-regulated during DT re-induction following the same strategy described earlier for up-regulated genes (**Supplementary Figure 1**). Comparative transcriptome analysis between R1 vs R1P revealed 3,728 genes whose expression is significantly reduced at R1P compared to R1 (adjusted *P* < 0.05; Benjamini–Hochberg method, log_2_ fold change < -1), and they were classified into four clusters based on expression values (Z-score of TPM) at R1, R1P, R5 and R5P samples using K-means cluster analysis (**Supplementary Figure 1A**; **Supplementary Table S5**). Genes in cluster 5 showed significant down-regulation only in R1P, the only DT tissues (*P* < 0.01, Steel–Dwass test) **(Supplementary Figure 1B**), indicating that genes from cluster 5 were negatively correlated to DT re-induction. This cluster 5 was made up by 103 genes, which in comparison to the 948 genes positively correlated to DT re-induction (previously described in **Figure 2** and **Supplementary Table S1**), implies that proportionally few genes were shut down to allow DT re-acquisition. A GO enrichment analysis for the cluster 5 genes (**Supplementary Figure 1C**) revealed that GO terms significantly enriched (FDR < 0.05, minimum number of mapped genes > 2) were mainly related to response to toxic substance and detoxification, in which, for instance, two peroxidase genes (*MtrunA17Chr2g0292111* and *MtrunA17Chr5g0404721*) and two alcohol dehydrogenase genes (*MtrunA17Chr3g0125911* and *MtrunA17Chr3g0125961*) were annotated. Further studies will be needed to clarify the causal relationship between the suppression of these detoxification-related genes and the re-induction of DT at R1P.

Additionally, the chromatin accessibility of cluster 5 genes was visualized by plotting of TSS enrichment scores based on the ATAC-Seq data (**Supplementary Figure 1D**) and, interestingly, no significant difference was observed for the chromatin accessibility of cluster 5 genes between R1, R1P and R5 samples (**Supplementary Figure 1E**), suggesting that expressional level of genes negatively correlated with the DT re-induction were not regulated at the chromatin level, suggesting a more direct transcriptional regulation of these genes.

To extend our analyses, we performed a genome-wide comparative ATAC-Seq analysis and detected 368 gene regions that were significantly more opened in DS (R1&R5) than DT samples (R1P) (**Figure 5A**; **Supplementary Table S6**). In other words, the chromatin state of these 368 gene regions was more condensed following a PEG treatment (R1P). Aiming to know the biological processes of genes showing a more condensed chromatin during DT re-induction, GO enrichment analysis of the 368 DS-(open)DARs (*i.e.*, DT-closed DARs) was performed (**Supplementary Figure 2A**). Interestingly, the most significantly enriched GOs were those related to signal transduction, even though the causal relationship between these signaling-related genes on DT re-induction was currently unknown. Finally, when comparing the 368 genes in DS-DARs identified using ATAC-seq analysis and the 103 genes belonging to cluster 5 detected by RNA-Seq experiments (**Supplementary Figure 2B**), no significant overlap between these two datasets was observed using Fisher’s exact test (*P* = 0.06, OR = 3.62), and only three genes were detected as DS-(open)DAR-DEGs (**Supplementary Table S6**). This result validated our previous observation that the transcriptional regulation of cluster 5 genes, whose expression was specifically repressed during DT re-induction, was not regulated at the chromatin level.

## Discussion

### Chromatin dynamics associated with DT re-induction in germinated seeds

**Figure 8:**
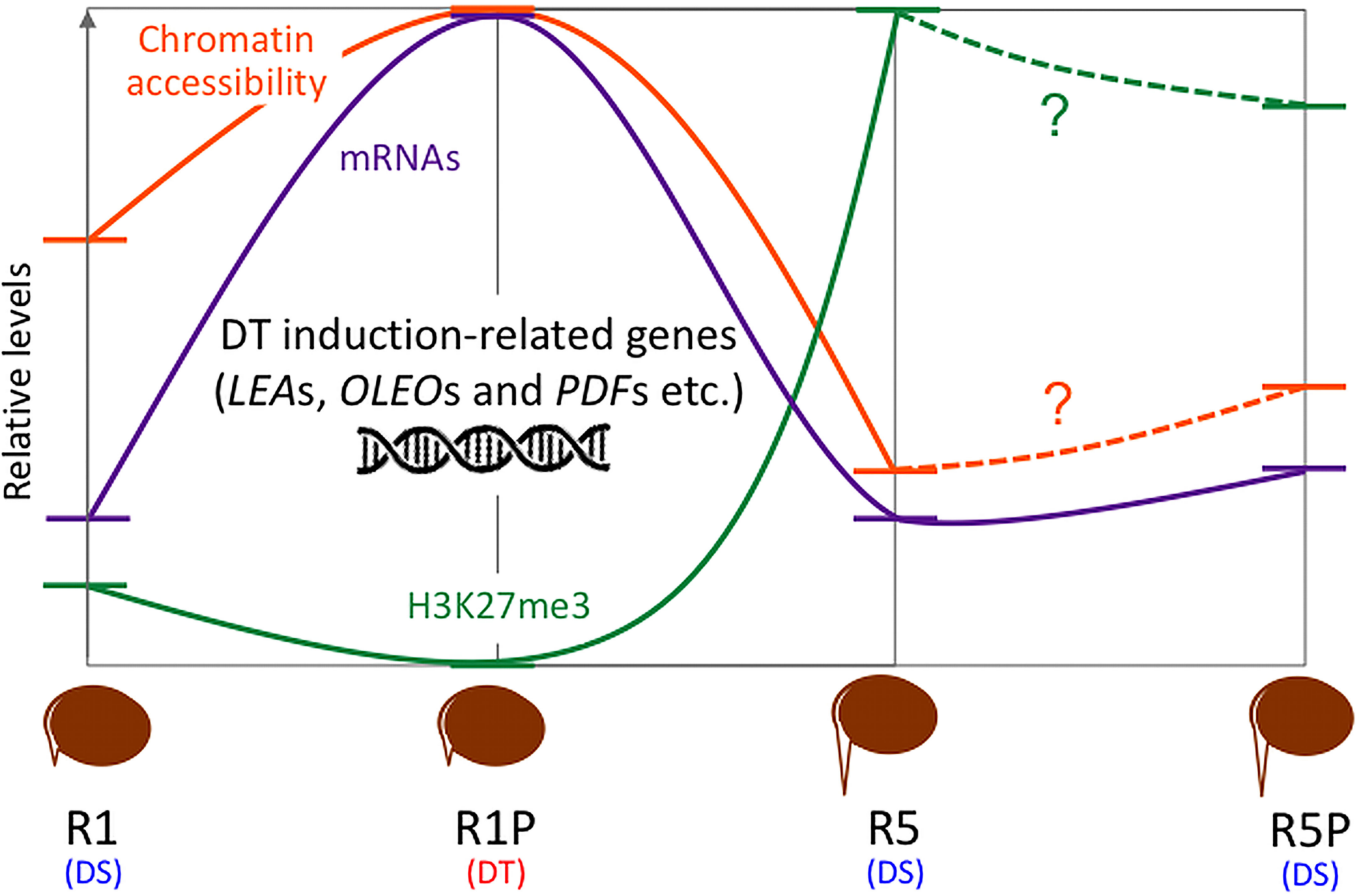
Comparative gene ontology (GO) enrichment analysis of DT-DAR-DEGs and DT-nonDAR-DEGs. Significant GO terms (FDR < 0.05, minimum number of mapped gene > 2, Fold change > 10) in “biological process” were obtained. Fold change represents fold enrichment of genes with the given term as compared to ones in the Medicago genome background. The number in the parentheses indicates the number of each DEG in cluster 1.

DT has been a key feature to conquer dry land for plants, being almost common in seeds but rare in vegetative tissues of angiosperms. After seed germination, plants undergo an irreversible transition from embryo to seedling development, accompanied by repression of embryonic traits and emergence of vegetative tissue. The DT is associated with an embryonic process, which is silenced after seed germination, firstly re-inducible following stimulation such as an osmotic stress during the DT window (Dekkers et al., 2015), then permanently silenced afterwards. In this study, we focused on the chromatin accessibility related to the DT inducibility before and after the DT window by analyzing the isolated radicles of germinated seeds in Medicago as materials, which displayed a loss of DT earlier than in the cotyledons. As described in the literature, in Medicago, the DT was re-inducible at an early germinating step corresponding to seeds with 1 mm radicle length seeds (R1) but not later at 5 mm radicle seed (R5) (**Figure 1**). RNA-Seq analyses revealed 948 genes in cluster 1 whose expression was specifically increased in R1P (**Figure 2**; **Supplementary Table S1**). Since R1P corresponds to the stage that tolerates desiccation following the PEG treatment (**Table 1**), we concluded that the cluster 1 includes important genes for re-induction of DT after germination. To date, ATAC-Seq analyses have previously demonstrated a positive correlation between gene chromatin accessibilities and expressional levels in many plant species (Wilkins et al., 2016; Tannenbaum et al., 2018; Dai et al., 2022). Similarly, our present study showed that cluster 1 genes have the highest accessibility to their chromatin in the R1P sample (**Figure 3**). Moreover, cluster 1 genes exhibited a significant decrease in their accessibility at R1, and even more at R5 (**Figure 3B**). These results suggested that (i) the pattern of gene expressions associated with DT re-induction after germination is regulated at the chromatin level with a change in the chromatin openness of those genes, and (ii) their chromatin regions are more closed in DS than DT radicles (**Figure 8**). At the opposite, when focusing on genes negatively correlated to DT re-induction (*i.e*. more expressed in R1 and R5 than in R1P samples), we identified 103 genes, that did not show a change in their chromatin openess between DT and DS samples, suggesting a more direct transcriptional regulation of these genes, without involvement of chromatin-related regulation (**Supplementary Figure 1A**; **Supplementary Table S5**). ChIP-Seq analysis for histone marks H2AK119ub demonstrated no clear signal enrichment on cluster 1 genes (**Figure 4A**), suggesting that PRC1 may not contribute significantly to repression of expression or chromatin closure of genes potentially involved in the re-induction of DT during the DT window. The gene expressions and chromatin accessibilities belonging to cluster 1 were clearly linked to H3K27me3 marks mediated by PRC2 (**Figure 4B**). Prominent H3K27me3 marks were observed especially in the R5 sample (**Figure 4C**), suggesting that these DT re-induction-related genes are targeted to be significantly repressed in their expression after the DT window during the post-germination developmental program due to this repressive histone mark (**Figure 8**). The repressive mark H3K27me3 is deposited on chromatin, covering thousands of genomic loci, including many developmental and stress-responsive genes in plants (Shen et al., 2021). For seed life cycle, H3K27me3 marks are involved in such as endosperm development, seed size, dormancy and somatic embryogenesis/callus formation after germination by contributing to repression of key regulatory gene expressions for each trait (Ding et al., 2022). As we detected prominent H3K27me3 marks in the DT re-induction related gene in R5, which is after the DT window (**Figures 4B, C**), it is possible that the seed DT switch is also regulated by the expression levels of genes through the H3K27me3 modifications. Further analysis focusing on effect of PEG treatment on the chromatin dynamics at the later stages of post-germination programs will be required to corroborate our hypothesis, such as the comparative chromatin modifications between R5 and R5P samples as well as relevant mutant functional analyses.

### Biological processes for DT re-induction *via* chromatin dynamics

The network of gene expressions involved in seed DT has been shown to overlap between acquisition during seed development and re-induction after germination (Terrasson et al., 2013; Costa et al., 2015), although its regulatory mechanism is not fully characterized. Present study revealed 88 DT-DAR-DEGs showing both chromatin openness and transcript levels markedly increased and being associated with DT re-induction (**Figure 5B**), which were annotated as involved in seed developmental processes such as seed maturation and dormancy (**Figure 6**). In parallel, the 860 DT-nonDAR-DEG, whose expressions were up-regulated similarly to aforementioned 88 DEGs but located in non-differentially accessible chromatin regions, showed over-representation of functional classes very distinct from those of the 88 DT-DAR-DEGs. This result suggests that chromatin dynamics of germinating seeds can specifically reprogram specific developmental processes for re-induction of DT following cues from external environment (*e.g.*, mild osmotic stress). Indeed, we showed that many gene functions are transcriptionally activity during the re-induction of DT, but the ones related to seed maturation were accompanied by the re-openness of their specific chromatin regions. This observation of differential dynamics of specific chromatin regions has already been reported in plants with specific and central regulatory gene regions in response to various environmental stresses such as phosphate limitation, drought, and cold stresses (Yang et al., 2020; Barragán-Rosillo et al., 2021; Ren et al., 2021; Wang et al., 2022).

Accumulation of LEAs is a landmark of seed maturation. The LEAs are known to be in an unstructured conformation at the hydrated state cells, whereas they readily adopt a more structured conformation upon drying. Due to their hydrophilic nature and hydration buffer capacity, certain LEAs are likely to have multiple functions in stress responses including desiccation. In *Medicago truncatula*, 12 seed-specific LEAs have been reported (Leprince et al., 2017), of which we detected the three LEAs (*MtEM6*, *MtPM25* and *MtrunA17Chr8g0348171*) in the DT-DAR-DEG list (**Figure 7**; **Supplementary Table S3**). *In vitro* studies of MtEM6 and MtPM25 (Boudet et al., 2006; Gilles et al., 2007; Boucher et al., 2010) demonstrated multifunctional protective capacities, such as enzyme protection, anti-aggregation against thermo-mechanical stress, and water binding. Consistent with this, Arabidopsis *em6* mutants showed defects in maturation drying (Manfre et al., 2009), suggesting that these LEAs in DT-DAR-DEGs could play important roles in DT re-induction after germination.

Four genes (*MtrunA17Chr1g0196841*, *MtrunA17Chr6g0449621*, *MtrunA17Chr2g0307331*, and *MtrunA17Chr3g0139191*) encoding oleosins, another typical protein involved in seed maturation, were also found as DT-DAR-DEGs in this study. Oleosins are predominant structural proteins in oil bodies that are largely seed-specific lipid organelles storing and protecting triacylglycerols, which are used as an energy source for germination and early plant growth. Consistent with our finding, it has been reported that lipid storage functional class were the most over-represented GO category associated with DT induction immediately after germination in Arabidopsis, and the main genes present in this GO category were oleosins (Costa et al., 2015). Oleosins prevent the lipid bodies from coalescing on dehydration and prevent the disruption of cellular structures during rehydration (Leprince et al., 1998; Pammenter and Berjak, 1999). They are also associated with freezing tolerance of seeds, probably by protecting integrity of the oil body membrane from freezing injury that are caused by multiple stresses, including cold, mechanical and severe dehydration stresses (Shimada et al., 2008). Moreover, interestingly, these seed DT-related proteins such as oleosins and LEAs have been shown to be co-opted for vegetative desiccation in resurrection plants (Costa et al., 2017; VanBuren et al., 2017). Future integrated studies on the chromatin dynamics of these seed-specific DT-related genes for diverse plant materials, such as maturating seeds, spores, and vegetative parts of resurrection plants, will be important for understanding the molecular mechanism of cellular/tissue-specific DT induction in plants.

Defense response-related GO terms were enriched from the DT-DAR-DEGs (**Figure 6**), in which three defensins (*MtPDF32*, *MtPDF33* and *MtPDF51*) were detected (**Supplementary Table S3**). Plant defensins are small, highly stable, cysteine-rich peptides that were, initially, identified as part of the innate immune system primarily directed against fungal pathogens. Most plant defensins and defensin-like peptides are relatively abundant in seed tissue, which could protect seed from soil fungi and thus enhance seedling survival rate (Stotz et al., 2009). However, defensins are expressed in almost every organ of plants in response to developmental stages and environmental stresses, suggesting that they may also act more broadly as ligands for cellular recognition and signaling beyond their presumed role in plant defense against fungal pathogens, although many of their molecular mechanisms have not yet been elucidated (Stotz et al., 2009; Sher Khan et al., 2019; Contreras et al., 2020). As an example, Arabidopsis exogenously treated with *Medicago sativa* defensins (MsDEF1 and MtDEF2) inhibited the of root and root hair growth (Allen et al., 2008). Recently, it was also reported that overexpression of Chickpea defensin gene *Ca-AFP* confers tolerance to water deficiency in Arabidopsis (Kumar et al., 2019). A detailed functional analysis of these genes would be needed to determine how defensins found in this study could be involved in the re-induction of DT after seed germination.

It is widely known that the regulation of seed maturation mostly relies on the network of the LAFL transcription factor network through *LEAFY COTYLEDON 1* (*LEC1*), *ABA-INSENSITIVE 3* (*ABI3*), *FUSCA 3* (*FUS3*) and *LEAFY COTYLEDON 2* (*LEC2*) genes. LAFL genes tightly control expression of many genes allowing proper seed development and maturation processes such as embryogenesis, suppression of germination, accumulation of storage proteins and acquisition of DT (Lepiniec et al., 2018; Smolikova et al., 2020). In Arabidopsis, *lec1*, *abi3* and *fus3* mutants, except for *lec2* are sensitive to desiccation (Keith et al., 1994; Meinke et al., 1994; To et al., 2006). None of these orthologues were however detected as DT-DAR-DEGs in this study. This indicates that LAFL genes, even if essential in the DT acquisition, may not be regulators of the DT re-induction between R1 and R1P at the post-germination phase of *Medicago truncatula*. The vegetative DT in the resurrection plant has been shown to not evolve through reactivation of the seed canonical LAFL network, suggesting that reactivation of components of the seed desiccation program in the vegetative tissues likely involves alternative transcriptional regulators (Lyall et al., 2020). In our study, we found ten genes encoding TFs as DT-DAR-DEGs, including *MtABI4*, an Arabidopsis ERF/AP2 transcription factor *ABI4* orthologue. ABI4 is known to regulate many aspects of plant development and stress responses through the ABA signaling, including seed maturation and germination (Wind et al., 2013). Direct evidence for involvement of ABI4 in the post-germination DT window has been provided by ABA signaling mutants analysis, in which *abi3*, *abi4* and *abi5* mutants were not able to re-establish DT of post-germination (Maia et al., 2014). The same study also observed a stronger reduced capacity to re-establish DT in *abi4* as its phenotype was already visible even at early stage of the DT window. These suggest that ABA signaling through MtABI4 may play important roles for re-establish DT of post-germination in *Medicago truncatula*, and *MtABI4* gene expression was shown to be controlled by its chromatin dynamics, although it was not linked to the level of H3K27me3 (**Figure 7B**). Moreover, two Arabidopsis orthologue TFs were identified in the DT-DAR-DEG list and have been reported as key regulators for seed germination, *AtSOM* and *AtGATA*. The CCCH-type zinc finger protein SOMNUS (SOM) was shown to integrate both light and high-temperature signals (Sano and Marion-Poll, 2021). In the dark, bHLH transcription factor PHYTOCHROME-INTERACTING FACTOR1 (PIF1) interacts with ABI3 and both bind to the promoter of *SOM* gene, which indirectly regulates ABA metabolism genes, resulting in an increase in ABA levels and inhibition of germination (Park et al., 2011). Similarly, at high temperature, both ABI3 and bZIP transcription factor ABI5 form a complex with the GA-signaling proteins DELLA and bind to the *SOM* promoter to activate its expression (Lim et al., 2013). The other Arabidopsis orthologue, the GATA-type zinc finger transcription factor *GATA12* is one of the downstream targets of DELLA protein RGA-LIKE2 (RGL2), which is a key transcriptional repressor of GA signaling. Freshly harvested seeds of *GATA12* suppression lines have reduced dormancy compared with the WT, while ectopic expression lines showed enhanced dormancy, indicating that GATA12 contributes to maintenance of primary seed dormancy (Ravindran et al., 2017). As both *SOM* and *GATA12* are repressive regulators of Arabidopsis seed germination, *MtSOM* and *MtGATA* may function as positive regulators to re-induce DT by re-programming the transition from post-germinated seeds to seedlings, in which chromatin dynamics with H3K27me3 may be involved (**Figure 7B**).

## Conclusion

In the present study, we captured the chromatin dynamics associated with seed desiccation tolerance/sensitivity at early germination in *Medicago truncatula* and revealed characteristic changes for a gene cluster involved in the DT re-induction. We observed changes in chromatin dynamics of genes encoding proteins with relevant biological functions such as LEAs, oleosins, defensins and several transcriptional factors during the DT re-induction. Even if we were not able to identify what regulates this dynamic during the re-induction during the DT window, we clearly highlighted the increase of histone mark H3K27me3 at the end of the DT window, which likely represses the DT program at this more advanced stage after germination. The expression sites of desiccation tolerance-related genes in land plants is mainly limited to their reproductive structures such as seeds and spores, except for specialized plants including resurrection plants. Our data not only update the information regarding regulation mechanisms of existing processes important for seed desiccation tolerance but also provide clues to activate the DT regulatory networks to severe dehydration in vegetative parts of plants that may contribute to minimize crop yield losses under drought stress.

## Methods

### Biological material

*Medicago truncatula* A17 plants were grown under standard conditions (20°C/18°C, 16 h photoperiod) in a controlled growth chamber. Plants were sown in round pots (25 cm). Seeds were collected at maturity stage, about 48 days after pollination (DAP) and were equilibrated at 44% of relative humidity (RH), using a saturated solution of K_2_CO_3_ at 20°C for 3 days, and then used for the subsequent analyses.

### Physiological analysis

The seed germination was accessed by germinating triplicates of 50 dried seeds on Whatman paper No1 imbibed with 1 ml of autoclaved water in 3 cm diameter Petri dishes at 20°C under a 16 h/8 h photoperiod for eight days. Then, seeds exhibiting a 1 mm (called 1mmD sample) and 5 mm (5mmD sample) radicles were sampled and used to perform a DT assay (Buitink et al., 2003), which consisted in slowly desiccating germinating seeds using a saturated solution of K_2_CO_3_ at 20°C for 72 hours. In parallel, 1 mm and 5 mm radicle seeds were subjected to a mild osmotic stress (-1.7 MPa) using a polyethylene glycol (PEG8000) solution for 3 days at 10°C dark (called 1mmPD and 5mmPD samples), then were washed and underwent desiccation as previously described. DT (re)induction capacity was tested for all sets of seeds (1mmD, 1mmPD, 5mmD, 5mmPD) by measuring the survival rate (*i.e.*, percentage of germination) of early post-germinating seeds after desiccation after rehydration and incubation at 20°C at a 16 h photoperiod in Petri dishes as described earlier and during 7 days. Seeds that were able to recover after desiccation, displaying developed cotyledons and elongated roots were annotated as desiccation tolerant, while seeds displaying only developed cotyledons but undeveloped roots were annotated as desiccation sensitive.

### Total RNA isolation and RNA-Seq

For Medicago early post-germination seeds, samples of radicles and cotyledons were used separately for RNA extractions. Samples were named accordingly: desiccated radicles of 1 mm germinating seeds were called R1 and R1P (when subjected to PEG treatment), desiccated radicles of 5 mm germinating seeds were called R5 and R5P (when subjected to PEG treatment). About 100 freshly harvested seeds in two biological replicates were used to extract RNA for each sample. All samples were ground using micropestles and liquid nitrogen and RNA was extracted using the NucleoSpin^®^ RNA Plant and Fungi kit (Macherey-Nagel, Düren, Germany) with lysis buffer containing 1% of polyvinylpyrrolidone (PVP-40) followed by incubation at room temperature for 10 minutes. RNA quantity and quality was measured using a NanoDrop ND‐1000 (NanoDrop Technologies, Wilmington, DE, USA) and RNA integrity using a 2100 Bioanalyzer (Agilent Technologies, Santa Clara, CA, USA). All samples with good qualities (260/280 and 260/230 absorbance ratio >1.8; RNA Integrity Number, RIN>7; 28S/18S>1.7) were sent to Beijing Genomics Institute (https://www.bgi.com) (Hong Kong) for library preparation and sequencing on BGISEQ-500 platform, generating an average of 24 M reads of 50 bp per sample (20M SE50). After quality control, high quality reads were mapped on *Medicago truncatula* (Mtv5) reference transcriptome of Mtv5 (annotation r1.6) using quasi-mapping alignment and quantification methods of Salmon algorithm v.1.2 (Patro et al., 2017). For gene expression analysis, raw RNA-Seq data were first normalized as Transcripts Per Kilobase Million (TPM). Differentially expressed genes (DEGs) were determined using DESeq2 package (v1.22.2) (Love et al., 2014) in RStudio (v1.3.1073)​​, with a threshold of *P*-adjusted value <0.05 for multiple testing with the Benjamini-Hochberg procedure which controls false discovery rate (FDR). K-means cluster analysis was performed with Pearson’s correlation distance by using MeV (v4.8.1) (Saeed et al., 2003). The RNA-seq data have been deposited in NCBI’s Gene Expression Omnibus and are accessible through GEO Series accession number GSE214468 (https://www.ncbi.nlm.nih.gov/geo/query/acc.cgi?acc=GSE214468).

### ATAC-Seq

The ATAC-seq experiment followed ENCODE experiment guidelines (https://www.encodeproject.org). Extraction of nuclei from Medicago radicles was adapted from Sikorskaite et al. (2013). About 30 radicles were used for each replicate at each stage, three biological replicates were used for R1 and R1P samples, and two biological replicates for R5 were used. Samples were initially grinded into a fine powder by an automatic grinder Tissue Lyser QIAGEN (Hilden, Germany) for 30 s at 25 hertz frequency with metal beads. 10 ml of cold nuclei isolation buffer (MES-KOH 10 mM pH 5.4, NaCl 10 mM, KCl 10 mM, EDTA 2.5 mM, Sucrose 250 mM, Spermidine 0.5 mM, Spermine 0.2 mM and DTT 1X) were added to each sample. Samples were then filtered through 70 µm, then 40 µm filters. Flow-through solutions were centrifuged at 1,200 × g for 10 minutes at 4°C. pellets were resuspended in 1 ml of cold Nuclei extraction buffer (sucrose 0.25 M, Tris-HCl 10 mM pH 8, MgCl_2_ 10 mM, Triton 1% and RCPI 1X), then centrifuged at 12,000 × g for 10 minutes at 4°C. The resulting pellet was resuspended in 300 µL of nuclei isolation buffer with 1.5 M sucrose concentration to separate nuclei by a sucrose gradient. Samples were centrifuged at 16,000 × g for 10 minutes at 4°C and the final pellet was resuspended in 1 ml of nuclei isolation buffer. Nuclei isolation was confirmed by microscopy with DAPI solution and nuclei amount was measured using a Neubauer cell (0.100 mm deep and 0.0025 mm^2^). 50,000 nuclei for each sample were used in the subsequent steps. Treatments with the hyperactive Tn5 transposase, DNA purification and library generation were performed using the Active Motif ATACseq kit as described by the manufacturers. Samples were sent for Beijing Genomics Institute (https://www.bgi.com) (Hong Kong) for sequencing at least 60 M reads of 100 bp paired-end (PE100). Raw reads were filtered to remove adapters and low-quality reads, then mapped to *Medicago truncatula* reference transcriptome (version 5) (Pecrix et al., 2018). Quality control was performed using FastQC and MultiQC algorithms (Andrews, 2010; Ewels et al., 2016). STAR algorithm (Dobin et al., 2013) was used to map paired-end reads to the *Medicago truncatula* reference genome. Deduplicated reads were first marked using Picard MarkDuplicates (https://gatk.broadinstitute.org/hc/en-us/articles/360037052812-MarkDuplicates-Picard) then removed using Samtools (Li et al., 2009). Differentially accessible regions (DARs) were identified using BAMscale (Pongor et al., 2020) to generate a count file of genomic regions comprising 1.5 Kb promoter and coding regions based on genome annotation followed by DESeq2 to statistically revealed DARs with a threshold of *P*-adjusted value < 0.05 for multiple testing with the Benjamini-Hochberg procedure which controls false discovery rate (FDR). The GO enrichment analysis was carried out using a web-based platform: ShinyGO (v0.76.2) (Ge et al., 2020), applying FDR cut-off < 0.05, minimum number of mapped gene > 2 and fold enrichment of gene > 10. The DARs were annotated using Bedtools Intersect (Quinlan and Hall, 2010) as being located in 1 Kb promoter regions and/or gene coding regions based on genome annotation. Bigwig files were generated using BamCoverage followed by BAMscale for normalization. The ATAC-Seq data have been deposited in NCBI’s Gene Expression Omnibus and are accessible through GEO Series accession number GSE214221 (https://www.ncbi.nlm.nih.gov/geo/query/acc.cgi?acc=GSE214221).

### ChIP-Seq

The ChIP-seq experiments followed ENCODE experiment guidelines (https://www.encodeproject.org). Chromatin immunoprecipitation and purification protocols were modified from (Cortijo et al., 2018) and adapted to seed tissues and described in Malabarba et al. (2022). Around 700 Medicago seed radicles (300 mg of dry weight) were used for each replicate at each stage and/or treatment. Two biological replicates were used for each sample for R1, R1P and R5. Harvested samples were ground into fine powder. 20 ml of crosslink buffer (0.4 M sucrose, 10 mM Tris-HCl pH 8, 1 mM EDTA, 1% formaldehyde, Cocktail protease inhibitor) was added to each sample and incubated at room temperature for 10 minutes. 2 ml of 2M Glycine was added and incubated for 5 minutes to stop the reaction. Crosslink buffer was removed by centrifugation (5 minutes at 3,000 × g). Nuclei isolation buffer (0.25 M sucrose, 15 mM PIPES pH 6.8, 5 mM MgCl_2_, 60 mM KCl, 15 mM NaCl, 1 mM CaCl_2_, 0.9% Triton-X100, Cocktail protease inhibitor) was added to samples and incubated on ice for 30 minutes. Samples were filtered on 70 µm, then 40 µm filters and flow-through solutions were centrifuged at 11,000 × g for 20 minutes at 4°C. Pellets were resuspended in 1 ml of Nuclei lysis buffer (50 mM HEPES pH 7.5, 150 mM NaCl, 1 mM EDTA, 1% SDS, 0.1% sodium deoxycholate, 1% Triton X-100, Cocktail protease inhibitor). Samples were then sonicated for 15 minutes with pulses of 75 W using a Sonicator Covaris M220. Samples were, then, centrifuged (13,800 × g for 10 minutes at 4°C) and supernatants were collected. 100 µl of the supernatant was stored at -80°C to serve as INPUT samples, while 600 µl were used for IP samples. IP samples were diluted 10 times with the nuclei lysis buffer without SDS. Samples were incubated with 10 µl of magnetic beads (5 µl Dynabeads^®^ Protein G/5µl Dynabeads^®^ Protein A) for 2 hours at 4°C with gentle agitation. Then, 20 µl of washed magnetic beads were added per IP samples (10 µl Dynabeads^®^ Protein G/10 µl Dynabeads^®^ Protein A) and 3 µg of antibody (either H3K27me3 or H2AK119Ub) was added to 250 µl of different samples. Samples were, then, incubated overnight at 4°C with gentle agitation for immunobinding of H3K27me3 and H2AK119Ub antibodies (Active Motif, Carlsbad, California, United States). Sample purification followed multiple washes using magnetic beads and the following buffers: low-salt washing buffer (150 mM NaCl, 20 mM Tris-HCl pH 8.0, 0.2% SDS, 0.5% Triton-X100 and 2 mM EDTA); high-salt washing buffer (500 mM NaCl, 20 mM Tris-HCl pH 8.0, 0.2% SDS, 0.5% Triton-X100 and 2 mM EDTA); LiCl washing buffer (0.25 M LiCl, 1% sodium deoxycholate, 10 mM Tris-HCl pH 8, 1% NP-40 and 1 mM EDTA) and TE buffer (1 mM EDTA and 10 mM Tris-HCl pH 8). Samples were resuspended in the elution buffer at 65°C (0.5% SDS and 0.1 M NaHCO_3_). Decrosslinking was performed on IP and INPUT samples by adding 0.5 µl 5 M NaCl per 10 µl of sample and incubated at 65°C overnight. Samples were purified by adding 10 µl of 0.5 M EDTA, 20 µl of Tris-HCl pH 6.5, 1 µl of RNase A and incubating for 30 minutes at 42°C then adding 1 µl proteinase K (20 mg/ml) and incubated for 1.5 hours, 45°C to digest proteins. Equilibrated AMPure beads (Beckman Coulter, #A63880, CA, USA) were used for sample washing (2 (beads): 1 (sample) ratio). Finally, beads were resuspended in 25 µl of Tris-HCl 10mM pH8 and supernatant was used for library preparation. DNA concentration was measured by Qubit™ dsDNA HS Assay Kit (Thermo Fisher Scientific, Waltham, Massachusetts, United States) and 50 ng of purified chromatin were used as starting amount for PCR amplification. Libraries were constructed following MicroPlex Library Preparation Kit v2 High Performance Library Preparation for Illumina^®^ NGS Platforms (Diagenode SA. Liège, Belgium). Library products were evaluated by Qubit and fragment size were measured using a Bioanalyzer 2100 instrument (Agilent Technologies, Santa Clara, CA, USA). Samples were sent to Beijing Genomics Institute (https://www.bgi.com) (Hong Kong) for sequencing on BGISEQ-500 platform, generating at least 40 M reads of 100 bp paired-end per sample (PE100). The bioinformatic pipeline performed to analyze the ChIP-Seq data was the same as previously described to analyze ATAC-Seq data, except for the last part. “Peak calling” regions (*i.e.*, enriched regions associated with the histone marks) were identified using Macs2 callpeak function (Zhang et al., 2008) by comparing treatment (IP) versus control (INPUT) files, and these peaks were annotated using Bedtools Intersect (Quinlan and Hall, 2010) as being located in 3 Kb promoter regions and/or gene coding regions based on genome annotation. Then, bigwig files were generated using Macs2 bdgcmp function (Zhang et al., 2008) using the method ‘qPois’, then followed by bedgraph2bigwig from the UCSC genome utilities (https://genome.ucsc.edu/goldenPath/help/bigWig.html). The ChIP-seq data have been deposited in NCBI’s Gene Expression Omnibus and are accessible through GEO Series accession number GSE214218 (H2AK119Ub) and GSE214220 (H3K27me3) (https://www.ncbi.nlm.nih.gov/geo/query/acc.cgi?acc=GSE214218, https://www.ncbi.nlm.nih.gov/geo/query/acc.cgi?acc=GSE214220).

### Data plots and statistical analyses

Violin points were drawn using the package Plotly (v4.10.1) (Sievert, 2020) in R. The TSS enrichment scores were calculated from bigwig files of ATAC-Seq and ChIP-Seq using the ComputeMatrix program then the score was plotted as normalized read density using the plotProfile program in deepTools (v3.5.0) (Ramírez et al., 2016) at ±3 Kb of TSS for each gene cluster. The Venn diagram was generated using a web-based platform: eulerr (Larsson, 2021) and the overlap of two gene sets was evaluated by Fisher’s exact test. Significant differences of log_2_TPM value and normalized read density among three or more groups were evaluated by Steel–Dwass test. All statistical tests were performed using R.

## Data availability statement

The datasets presented in this study can be found in online repositories. The names of the repository/repositories and accession number(s) can be found below: https://www.ncbi.nlm.nih.gov/, GSE214221; https://www.ncbi.nlm.nih.gov/, GSE214218; https://www.ncbi.nlm.nih.gov/, GSE214220; https://www.ncbi.nlm.nih.gov/, GSE214468.

## Author contributions

NS, JM, ZC, DW, and JV performed experiments. NS, JM, SG, and JV analyzed data. NS and JV wrote the manuscript. All authors reviewed the manuscript.

## Funding

This research was part of the DEswitch project funded by ANR (ANR‐19‐CE20‐0027‐01).

## Acknowledgments

The authors sincerely thank Julie Marie Pelletier and John Harada (University of California Davis, USA) for their helpful advice regarding ChIP-seq experiments and all the SEED team at IRHS Angers, more specifically Joseph Ly Vu and Julia Buitink for sharing their experience with the early post germination desiccation assay on Medicago. The authors also gratefully acknowledge the technical platforms ANAN (Genomics) and iMAC (Microscopy) of the SFR 4207 QUASAV for their technical support.

## Conflict of interest

The authors declare that the research was conducted in the absence of any commercial or financial relationships that could be construed as a potential conflict of interest.

## Publisher’s note

All claims expressed in this article are solely those of the authors and do not necessarily represent those of their affiliated organizations, or those of the publisher, the editors and the reviewers. Any product that may be evaluated in this article, or claim that may be made by its manufacturer, is not guaranteed or endorsed by the publisher.

## Supplementary material

Supplementary Figure 1 Genes from cluster 5 whose expressions were negatively correlated with DT induction. **(A)** K-means cluster analysis of 3,728 genes whose expression is significantly down-regulated at R1P compared to R1 (adjusted *P* < 0.05; Benjamini–Hochberg method, log_2_ fold change < -1). The number in the parentheses indicates the number of genes in each cluster. Z-score of TPM values was used to normalize each gene expression level. **(B)** Violin plots showing log_2_TPM values of genes in cluster 5 at R1, R1P, R5 and R5P (**P* < 0.01, Steel–Dwass test, compared to R1). Dashed lines represent the mean value for each sample. **(C)** GO enrichment analysis of genes in cluster 5. Significant GO terms (FDR < 0.05, minimum number of mapped genes > 2) in “biological process” were represented. Fold change represents fold enrichment of genes with the given term as compared to the whole gene set annotated in Medicago **(D)** TSS enrichment scores from ATAC-seq were plotted at ±3 Kb of the transcription start site (TSS) of genes present in cluster 5. **(E)** Violin plots showing normalized read density of genes (1 Kb promoter + mRNA) in cluster 5 at R1, R1P and R5. Dashed lines represent the mean value for each sample. Different letters indicate significant differences (**P* < 0.01, Steel–Dwass test).

Supplementary Figure 2 Genes in open chromatin regions in DS samples. **(A)** GO enrichment analysis of genes in DS-DARs. Significant GO terms (FDR < 0.05, minimum number of mapped gene > 2) in “biological process” were represented. Fold change represents fold enrichment of genes with the given term as compared to the whole gene set annotated in Medicago. **(B)** Venn diagrams show overlaps between genes in DS-DARs detected by ATAC-Seq and genes belonging to cluster 5 detected by RNA-Seq. No significant overlap was detected (*P* values from Fisher’s exact test; OR: odds ratio which represents the strength of association, with OR>1 corresponding to a strong association).
