# Supplementary figures and images for: Chromatin dynamics associated with seed desiccation tolerance/sensitivity at early germination in Medicago truncatula
Source: Front Plant Sci. 2022 Nov 24;13:1059493. doi: 10.3389/fpls.2022.1059493 (PMC9729785; doi:10.3389/fpls.2022.1059493)

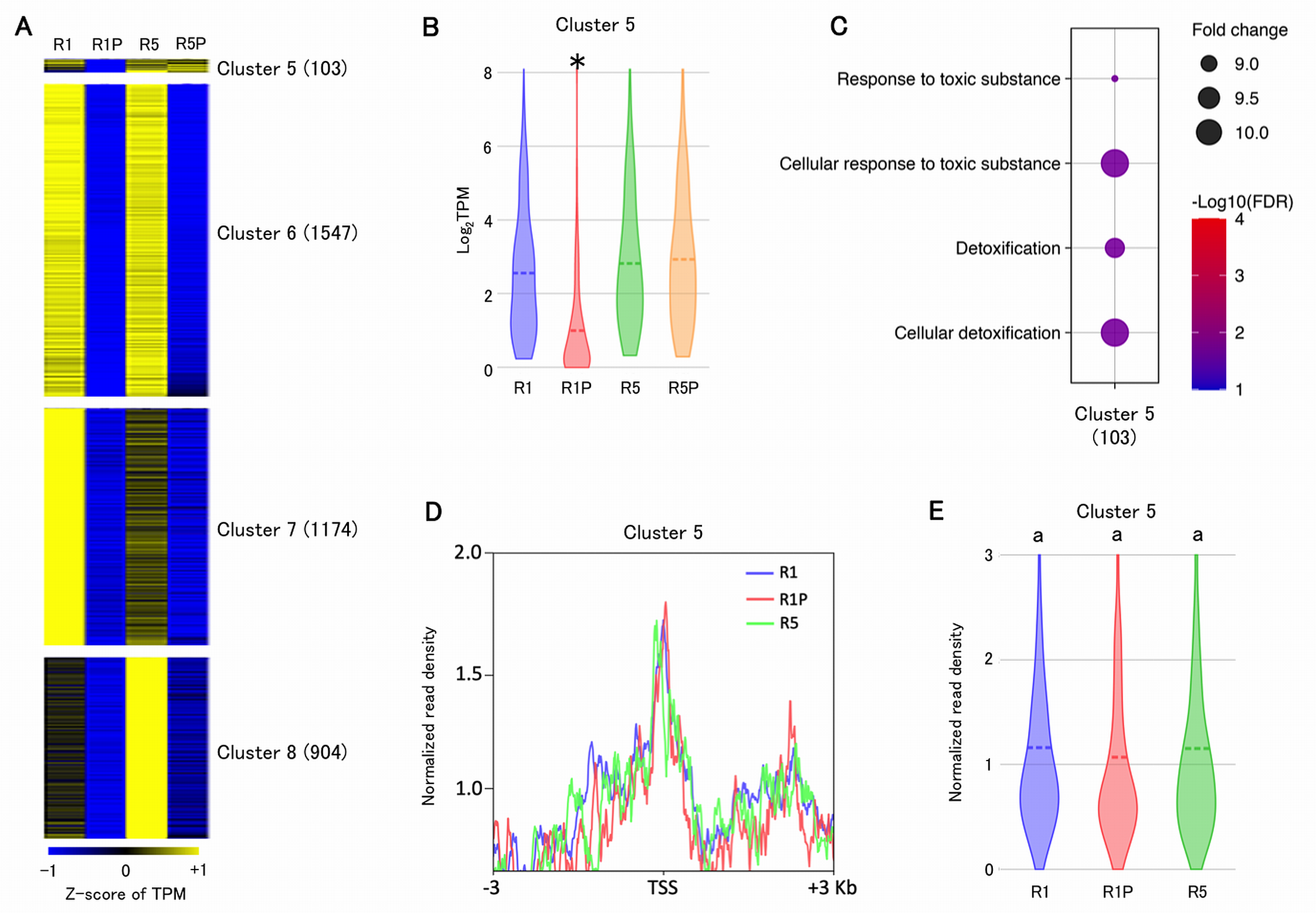

Supplement: Supplementary Figure 1 — Genes from cluster 5 whose expressions were negatively correlated with DT induction. (A) K-means cluster analysis of 3,728 genes whose expression is significantly down-regulated at R1P compared to R1 (adjusted P < 0.05; Benjamini–Hochberg method, log2 fold change < -1). The number in the parentheses indicates the number of genes in each cluster. Z-score of TPM values was used to normalize each gene expression level. (B) Violin plots showing log2TPM values of genes in cluster 5 at R1, R1P, R5 and R5P (*P < 0.01, Steel–Dwass test, compared to R1). Dashed lines represent the mean value for each sample. (C) GO enrichment analysis of genes in cluster 5. Significant GO terms (FDR < 0.05, minimum number of mapped genes > 2) in “biological process” were represented. Fold change represents fold enrichment of genes with the given term as compared to the whole gene set annotated in Medicago (D) TSS enrichment scores from ATAC-seq were plotted at ±3 Kb of the transcription start site (TSS) of genes present in cluster 5. (E) Violin plots showing normalized read density of genes (1 Kb promoter + mRNA) in cluster 5 at R1, R1P and R5. Dashed lines represent the mean value for each sample. Different letters indicate significant differences (*P < 0.01, Steel–Dwass test). [file Image_1.tif]

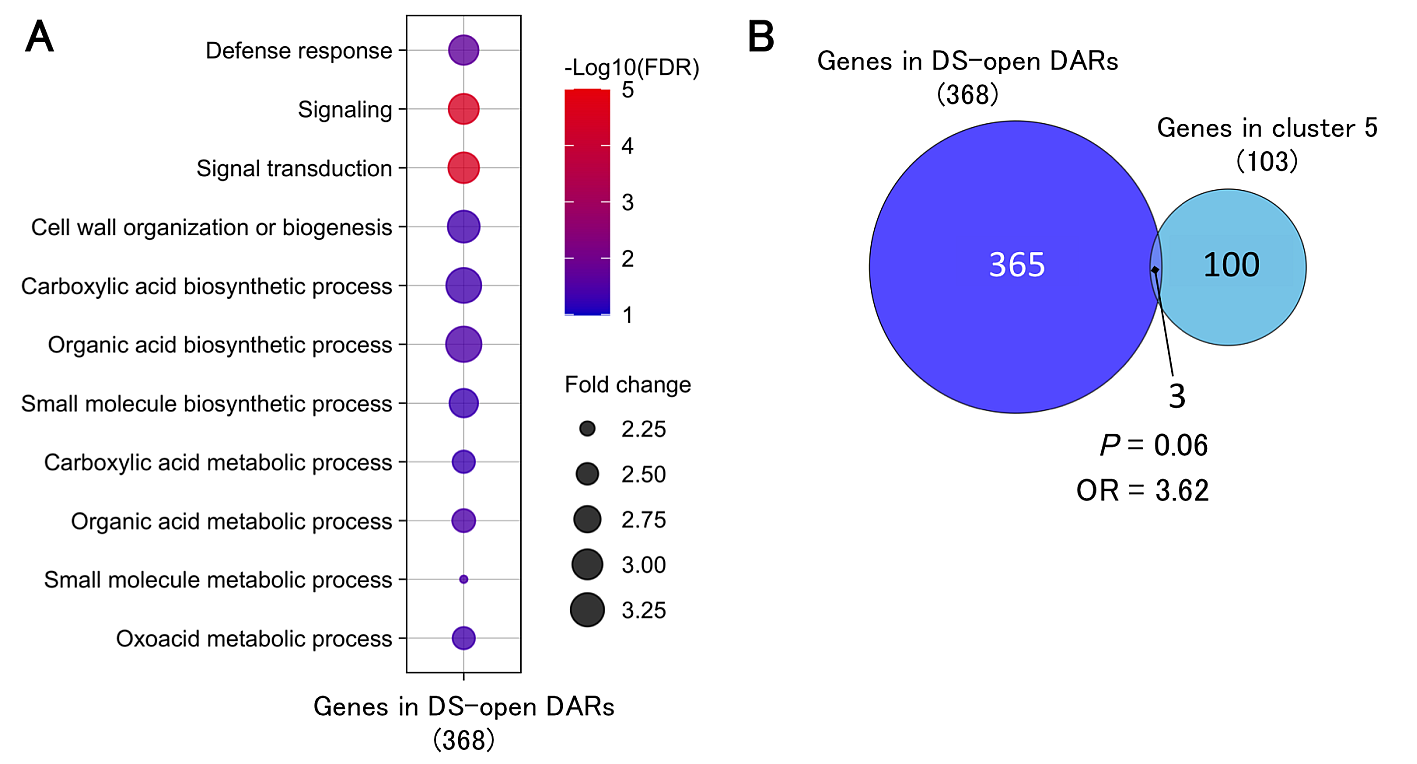

Supplement: Supplementary Figure 2 — Genes in open chromatin regions in DS samples. (A) GO enrichment analysis of genes in DS-DARs. Significant GO terms (FDR < 0.05, minimum number of mapped gene > 2) in “biological process” were represented. Fold change represents fold enrichment of genes with the given term as compared to the whole gene set annotated in Medicago. (B) Venn diagrams show overlaps between genes in DS-DARs detected by ATAC-Seq and genes belonging to cluster 5 detected by RNA-Seq. No significant overlap was detected (P values from Fisher’s exact test; OR: odds ratio which represents the strength of association, with OR>1 corresponding to a strong association). [file Image_2.tif]
